# Use of a food frequency questionnaire to assess diets of Jamaican adults: validation and correlation with biomarkers

**DOI:** 10.1186/1475-2891-10-28

**Published:** 2011-04-09

**Authors:** Maria D Jackson, Susan P Walker, Novie M Younger, Franklyn I Bennett

**Affiliations:** 1 Dept of Community Health and Psychiatry, University of the West Indies, Mona, Kingston, Jamaica; 2 Tropical Medicine Research Institute, University of the West Indies, Mona, Kingston, Jamaica; 3 Dept. of Pathology, University of the West Indies, Mona Campus, Kingston, Jamaica

## Abstract

**Background:**

Assessment of habitual diet is important in investigations of diet-disease relationships. Many epidemiological studies use the food frequency questionnaire (FFQ) to evaluate dietary intakes but few studies validate the instrument against biological markers. The aim of this study was to assess the validity and reproducibility of a previously validated 70-item food frequency questionnaire (FFQ) that was expanded to 120-items to assess diet - cancer relations.

**Methods:**

Relative validity of the FFQ was assessed against twelve 24-hour recalls administered over 12 months in 70 subjects. The FFQ was repeated after one year (FFQ2) to assess reproducibility. The validity of the FFQ was evaluated by comparing nutrient and food group intakes from 24-hour recalls with the first and second FFQ. In addition, FFQ validity for cholesterol and folate were determined through correlation with biomarkers (serum cholesterol, serum folate and whole blood folate) in 159 control subjects participating in a case-control prostate cancer study.

**Results:**

Compared to recalls the FFQ tended to overestimate energy and carbohydrate intakes but gave no differences in intake for protein and fat. Quartile agreement for energy-adjusted nutrient intakes between FFQ2 and recalls ranged from 31.8% - 77.3% for the lowest quartile and 20.8% - 81.0% in the highest quartile. Gross misclassification of nutrients was low with the exceptions of protein, vitamin E and retinol and weighted kappa values ranged from 0.33 to 0.64 for other nutrients. Validity correlations for energy-adjusted nutrients (excluding retinol) were moderate to high (0.38- 0.86). Correlation coefficients between multiple recalls and FFQ1 ranged from 0.27 (fruits) to 0.55 (red meat); the second FFQ gave somewhat higher coefficients (0.30 to 0.61). Reproducibility correlations for the nutrients ranged from 0.50 to 0.84.

Calibration of the FFQ with biochemical markers showed modest correlations with serum cholesterol (0.24), serum folate (0.25) and whole blood folate (0.33) adjusted for age, energy, body mass index and smoking.

**Conclusions:**

The expanded FFQ had good relative validity for estimating food group and nutrient intakes (except retinol and vitamin E) and was a reliable measure of habitual intake. Associations with biomarkers were comparable to other studies.

## Background

Accurate estimates of habitual dietary intake remain a challenge in the study of diet-disease relationships. Food frequency questionnaires (FFQs) have been used to assess long-term dietary intakes, an important exposure factor for conditions such as cardiovascular diseases and some cancers [1]. The use of food frequency questionnaires has been advantageous to assess diet since methods such as 24-hour recalls and food records do not reflect past diet or usual intake and are generally expensive [2].

Validation and calibration studies of FFQs are essential in nutritional epidemiology for the interpretation of findings and comparability between studies. Validation studies provide information on the extent to which the method actually measures what it was designed to measure, whereas calibration studies determine how one method of dietary assessment compares with a reference method [3]. Although FFQs are often validated against other methods of dietary assessment such as dietary records and dietary recalls, factors which affect the validity of the FFQ such as memory and nutrient data are also likely to influence the reference method. Random errors between the two methods cannot be assumed and could lead to higher estimates of correlation between the FFQ and the reference method [4]. Furthermore, underestimation of correlation could occur as a result of associated random errors in repeated measurements [4,5]. Biochemical markers have been used as surrogate markers of dietary intakes as the potential sources of random errors are different from errors in any dietary assessments [6] and are largely independent of measurement errors associated with memory [7]. Correlations between nutrient intake and biochemical markers would provide additional evidence of the validity of the FFQ.

In 2001, we developed a 70-item FFQ to assess energy and nutrient intakes of adult Jamaicans of African origin as part of a study of the epidemiology of diabetes and hypertension. The FFQ showed reasonable estimates of dietary intake when compared with twelve repeated 24-hour recalls over one year [8]. We have now expanded the list of food items to assess dietary intakes in general and diet and cancer relations in particular. In this report we present the validity of the expanded 120-item food frequency questionnaire compared with repeated 24-hour recalls (n = 12) over a one-year period and the reproducibility of the FFQ over one year. In addition we compare intakes assessed by the FFQ with nutritional biomarkers (cholesterol and folate) in cancer-free men enrolled in a case control study of diet and prostate cancer.

## Methods

The study had two components (i) comparison of the FFQ with repeated 24-hour recalls provided by 70 free living individuals residing in urban and rural areas and (ii) comparison of FFQ dietary intake data with biomarkers of diet obtained from control subjects (n = 159) participating in the case-control prostate cancer study.

The study was approved by the Ethics committee of the University of the West Indies and subjects gave written informed consent to participate in the study.

### i. Dietary intakes

#### Study population

Free living individuals (n = 100) residing in urban and rural communities identified by the Statistical Institute of Jamaica (STATIN) to be similar in socio-economic characteristic to the general population were recruited. Communities were defined as an enumeration district (ED). The STATIN map of the ED indicated the starting point and boundaries of the community. From the starting point, an adult from every second household was systematically selected to provide information on dietary intakes by means of 24-hour recalls and the revised FFQ. Using age categories of 25-39, 40-54 and 55 or more years, a sample of 100 persons was recruited divided into 6 age/sex categories. Participants were asked to provide twelve 24-hour recalls over a one-year period and to complete food frequency questionnaires at the beginning and end of the year. Twenty one participants (15 males and 6 females) who only partially completed the study were excluded from the analyses.

#### Dietary measurements - repeated 24-hour recalls

Three 24-hour recalls were administered on consecutive days to each participant at 3 month intervals, yielding a total of 12 recalls for each subject over one year (January - December, 2005). Recalls for each subject included all days of the week: 8 weekdays and 4 week-end days. One trained nutritionist collected data on 24-hour recalls and subjects were unaware of the days the interview would be conducted. The interviewer requested participants to recall all food and drink consumed over the previous 24 hours. Portions were carefully estimated by use of food models, household measures and utensils in conjunction with a detailed description of the food and method of preparation.

#### Expanded food frequency questionnaire

Habitual dietary intake was assessed by the expanded version of the previously validated questionnaire. The expanded version consisted of 120 food and drink items and was administered by interview. Whereas the original 70-item food frequency questionnaire was designed to investigate obesity, hypertension and diabetes, the objective of the revised version was to investigate adult diets with a focus on cancer. Food items added were based on hypothesized relationships with foods (e.g. expanded listing of peas, beans, vegetables, soy and soy products) and nutrients (including phytonutrients) with cancer.

Frequency of usual food consumption was estimated using one of 8 pre-coded categories of responses. For each food item, participants were asked to supply information on portion size by using food models, commonly used household utensils, measuring cups and a measuring tape to indicate, the portion size usually consumed. The FFQ was administered by interview by four trained research nurses at the beginning and end of the year during which recalls were measured. Subjects were asked to report their usual consumption pattern over the previous year.

Nutrient content of food items was calculated using largely the US Department of Agriculture Nutrient Database (USDA, 2007), other published sources, the University of the West Indies Chemistry and manufacturers laboratories.

Daily nutrient intakes were calculated from the questionnaire by multiplying the frequency of use by the nutrient composition specified for each food item and its portion weight, using a computer programme written for SPSS. In analysis, coefficients of 0.0, 0.03, 0.08, 0.14, 0.40, 0.80, 1.00 and 2.5 were used to indicate frequencies of almost never, once per month, 2-3 times per month, once per week, 2-4 times per week, 5-6 times per week, once per day and 2 or more times per day, respectively. Nutrients from all foods were summed to obtain a total nutrient intake for each individual.

### (ii). Biomarkers of cholesterol and folate - calibration study

#### Study population

The biomarker sample was a subset of subjects enrolled in the control group of a case-control study of diet and prostate cancer in Jamaica. The men enrolled in the validation study (n = 176) met the first level criteria for being a control in the study [prostate specific antigen (PSA) ≤ 2.0 μg/L or free / total PSA ≥ 25 μg/L]. The food frequency questionnaire was administered by trained research nurses to all subjects at enrollment to the diet and cancer study, March, 2005 - July, 2007.

#### Sample collection and biochemical analyses

Non-fasting venous blood samples (20 ml) were collected in the mornings and were placed on ice packs in a cooler before being taken to our laboratory in the afternoons. Whole blood aliquots of 5.0 ml were taken and the remaining blood samples were centrifuged at 800 revolutions per minute (rpm) for 10 min. and separated into aliquots of plasma and buffy coat fractions. Aliquots were stored at -70°C.

Total serum cholesterol was measured on the Abbott Architect analyser by a cholesterol oxidase method [9,10] with CVs of 3.28% for 6.4 nmol/L and 4.35 % for 2.6 nmol/L. Folic acid in whole blood and serum were measured on the Immulite analyser by a competitive immunoassay method. Measurement of cholesterol in serum and whole blood and serum folate was carried out at the Chemical Pathology laboratory of the University of the West Indies. Only two biomarkers of dietary intakes were investigated as resources were limited.

### (iii) Other measurements

Subjects in the biomarker investigation provided information on diet, socio-demographic factors, health behaviours, medical history, and use of medication and vitamins / supplements. Body mass index was calculated as weight (in kilograms) divided by height (in metres, squared) and weight status was classified according to the WHO classification.

### Exclusions

We excluded from analyses subjects whose reported energy intakes were implausible [outside the range of 800 to 5000 kcal (n = 9)]; the same criterion was applied in earlier reports of diet and disease relationships [11,12]. Men who were taking lipid-lowering drugs (n = 9), or supplements (n = 8) were excluded from biomarker analyses. A total of 70 participants were available for the dietary validation and reproducibility assessment and 159 men provided information on biological markers for the calibration of the FFQ.

### Statistical analyses

Data on 24-hour recalls and food frequency questionnaire were converted to nutrient intakes by a computerized dietary analysis system (Nutritionist V) [13]. Descriptive means and medians are presented on untransformed nutrients and food group data. Nutrients and biomarker variables were not normally distributed and both sets of variables were log transformed. Transformed variables were used for correlational analyses of nutrients.

Nutrient intakes were adjusted for total energy by computing residuals from regression analyses with energy intake as the independent variable and nutrient intake as the dependent variable [2]. Residuals were added to the expected nutrient value for the mean energy intake of the sample to obtain a score adjusted to the average energy intake. Pearson's product-moment correlation coefficients were computed to determine the associations between the FFQ and 24-hour recalls before and after adjustment for total energy intake. Spearman's correlation coefficients were applied for intakes of food groups from the FFQs and 24-hour recalls. Agreement and misclassification were expressed as the proportion of subjects classified, respectively, into the same and extreme quartiles of the distribution for a given nutrient intake. Weighted kappa statistic (K_w_)[14] values were calculated comparing quartiles of intake for each nutrient from FFQ1 and 24-hour recalls. The following values for kappa were used to evaluate agreement between the dietary methods: greater than 0.80 - very good agreement, 0.61-0.80 good agreement, 0.41 - 0.60 moderate agreement, 0.21- 0.40 fair agreement, and less than 0.20 poor agreement [14]. Intraclass correlation coefficients were used to provide estimates of the reproducibility of the FFQ.

Bland-Altman plots were employed to evaluate the agreement between the two dietary methods of assessment; the range of intakes was plotted against the average of the two methods. As recommended by Bland & Altman [15], nutrients were log (ln) transformed to narrow the limits of agreement (LOA). The antilogs of the point and 95% confidence interval estimates of the mean difference (bias) determined the ratio of the FFQ to 24-hour recall values, with 1.0 representing ideal agreement. In excess of 5% of the observations outside the 95% limits of agreement (mean bias ± 2sd) of the Bland-Altman plots suggest lack of comparability of the methods.

Men participating in the calibration study were classified into quartiles according to their intakes of cholesterol and folate as estimated from the FFQ. Mean values for cholesterol and folate biomarkers were obtained for each of their respective nutrient intake quartiles and they were analysed to determine evidence of a statistically significant trend across quartiles of intake. T-tests were used to assess differences between the means for the lowest and highest quartiles. Pearson's product-moment correlations were used to estimate crude associations between intakes (FFQ) and blood levels of cholesterol and folate. We obtained partial correlation coefficients for association between dietary intakes (FFQ) and biomarkers with adjustments for age, energy intake, body mass index, alcohol intake and current smoking as possible confounders that explain some of the variation in blood levels of the nutrients.

All analyses were performed using the Statistical Package for Social Sciences (SPSS) version 15.0 and Stata version 9. Statistical significance was achieved when p < 0.05.

## Results

The characteristics of subjects enrolled in the study are displayed in Table 1. The dietary arm of the study included both women and men and these participants were younger than the biomarker sample. Men in the dietary sample were fairly similar to the biomarker group with respect to height, body mass index (BMI) and the proportion of obesity.

**Table 1:** Characteristics of study participants enrolled in the expanded Jamaican reproducibility and validation / calibration ^a ^study

	Samples		
	**Dietary intakes**		**Biomarker ^a^**
	**Males**	**Females**	
***n***	**31**	**39**	**176**

Age (years): mean ± sd	40.9 ± 16.5	41.5 ± 16.8	61.6 ± 10.5^†^
Weight (kg): mean ± sd	77.9 ± 13.4	77.5 ± 20.6	74.8 ± 14.1
Height (m): mean ± sd	172.7 ± 6.6	161.6 ± 7.8**	171.6 ± 7.2
Body mass index (kg/m^2^)	26.1 ± 4.0	29.7 ± 8.1*	25.3 ± 4.4
Obesity (BMI≥30.0 kg/m^2^) (%)	14.3	34.2	14.8

^a ^Control population enrolled in the diet and prostate cancer study.

*p < 0.05; **p < 0.0001; genders significantly different

^† ^Males in biomarker and dietary samples significantly different.

### Relative Validity - dietary intakes

Mean energy and nutrient intakes from the twelve 24-hour recalls and two FFQs administered at the beginning and end of the one-year validation study are reported in Table 2. Intakes of energy, carbohydrate, and linolenic acid were significantly higher on both FFQs than the 24-h recalls, whereas dietary estimates of vitamin E was lower with the FFQs. Whereas the second FFQ when compared with the recalls underestimated fat intake, recalls were significantly higher for monounsaturated fat, polyunsaturated fat and linoleic acid. The remaining nutrients gave similar estimates on all 3 measurements.

**Table 2:** Mean daily nutrient intakes estimates of the two food frequency questionnaires (FFQs) and twelve 24-hour recalls, and correlations between the two dietary methods ^a^

				24-hour recalls *vs *FFQ 1^†^		24-hour recalls *vs*. FFQ 2^†^	
	Repeat 24- hour recalls	FFQ 1	FFQ 2	Unadjusted	Energy adjusted	Unadjusted	Energy adjusted
				r	***r***	***r***	***r***
Total Energy (kcal)	2324 ± 662	2541 ± 559*	2434 ± 644*	0.41		0.45	
Total Carbohydrate (g)	327.9 ± 105.8	361.1 ± 105.9*	340.3 ± 113.2*	0.37	0.35	0.38	0.38
Protein (g)	101.7 ± 29.9	91.4 ± 27.5	103.1 ± 36.5	0.26	0.25*	0.33	0.31
Total Fat (g)	66.2 ± 22.8	64.8 ± 23.1	63.5 ± 32.8*	0.38	0.41	0.37	0.40
Total Saturated Fat (g)	29.9 ± 11.9	25.5 ± 10.4	28.6 ± 11.8	0.45	0.48	0.42	0.43
Total MUFA (g)	19.8 ± 10.7	19.3 ± 8.5	21.9 ± 12.6*	0.51	0.59	0.40	0.46
Total PUFA (g)	12.5 ± 5.1	11.8 ± 4.4	13.3 ± 6.3*	0.33	0.29	0.36	0.38
Linoleic acid	15.7 ± 12.4	16.7 ± 14.2	16.1 ± 14.1	0.43	0.41	0.47	0.44
Linolenic acid	0.69 ± 0.2	0.81 ± 0.3*	0.73 ± 0.3*	0.22^‡^	0.35	0.38	0.43
Cholesterol (mg)	268.1 ± 106.1	259.7 ± 117.2	251.2 ± 114.0	0.52	0.53	0.47	0.41
Calcium (mg)	727 ± 218	782 ± 281	783 ± 341	0.44	0.50	0.45	0.45
Iron (g)	21.7 ± 9.3	20.0 ± 5.9	22.1 ± 9.8	0.43	0.42	0.44	0.43
Retinol (ug)	2171 ± 1680	2201 ± 1405	1860 ± 1143	0.21^‡^	0.22	0.18^‡^	0.17^‡^
Beta-carotene (mg)	2271 ± 2420	2410 ± 2680*	1983 ± 2256**	0.35	0.33	0.34	0.34
Vitamin E (mg)	19.7 ± 7.5	15.4 ± 7.8**	16.8 ± 6.1**	0.21^‡^	0.20^‡^	0.23	0.23
Folate (g)	625.7 ± 242	634 ± 233	590 ± 185	0.56	0.59	0.42	0.45
Total dietary fiber (g)	28.6 ± 11.7	33.7 ± 11.8	30.7 ± 11.7	0.38	0.44	0.43	0.50
Alcohol (g)	2.8 ± 5.4	2.4 ± 2.7	2.6 ± 9.1	0.75	0.79	0.85	0.86

^† ^All variables were log-transformed before analysis.

^‡ ^Not significant; all correlations are significant to *p*≤ 0.001, unless indicated otherwise * *p*≤ 0.05.

^a ^Energy-adjusted correlations between dietary methods as suggested by Willett (1)

* *p*≤ 0.05; ** *p*≤ 0.001 - mean FFQ intake significantly different from repeat recalls.

Pearson's correlation coefficients for unadjusted and energy-adjusted nutrient intakes estimated from each administration of the FFQ and 24-hour recalls are also presented in Table 2. Correlation coefficients between recalls and FFQ1 for unadjusted energy and nutrients ranged from 0.21 (vitamin E) to 0.75 (alcohol) and with FFQ2 from r = 0.08 (retinol) to 0.85 (alcohol). Energy-adjustment decreased the correlations between recalls and FFQ 1 and FFQ2 for protein, fat, linoleic acid and iron and inconsistently influenced polyunsaturated fat, cholesterol and retinol. For the other nutrients energy adjustment led to modest increases in correlation coefficients. Partial correlations between recalls and FFQs, controlling for age and body mass index, revealed coefficients that were similar to Table 2 (data not shown).

**Figure 1:**
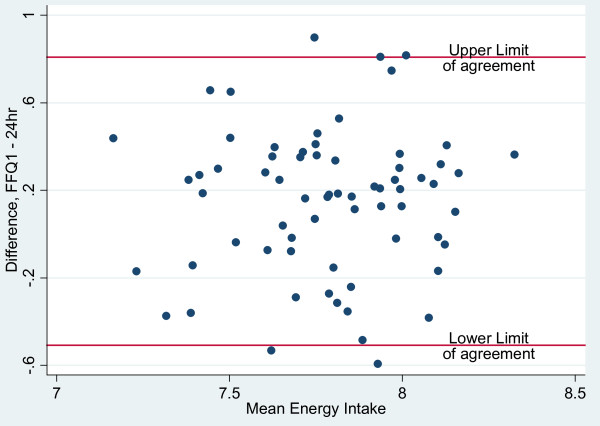
**Bland-Altman plots illustrating the level of agreement between dietary energy intake (log transformed data; n = 70) from FFQ 1 and 24-hour recall data for Jamaican adults**.

**Figure 2:**
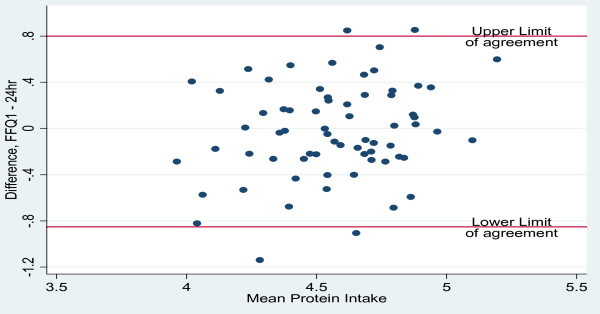
**Bland-Altman plots illustrating the level of agreement between protein intake (log transformed data; n = 70) from FFQ 1 and 24-hour recall data for Jamaican adults**.

**Figure 3:**
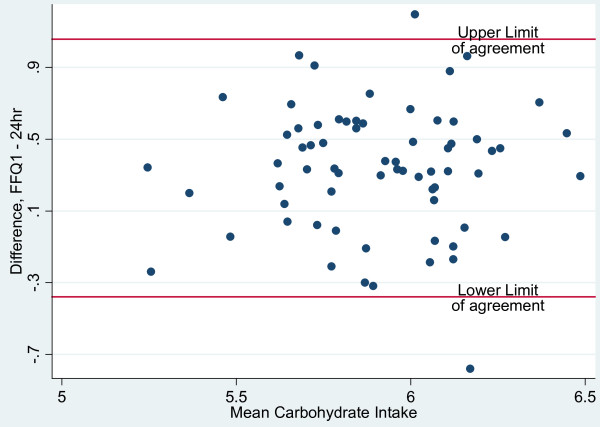
**Bland-Altman plots illustrating the level of agreement between carbohydrate intake (log transformed data; n = 70) from FFQ 1 and 24-hour recall data for Jamaican adults**.

**Figure 4:**
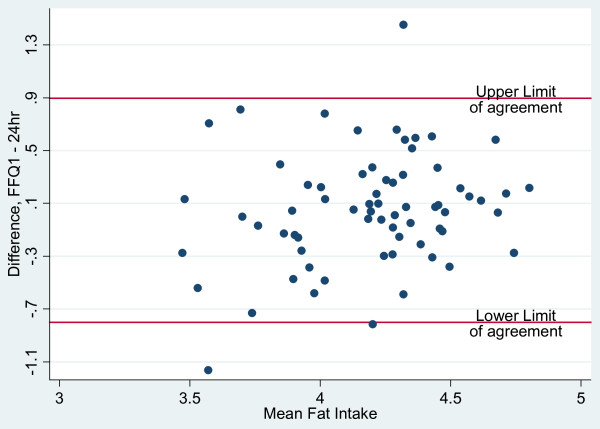
**Bland-Altman plots illustrating the level of agreement between fat intake (log transformed data; n = 70) from FFQ 1 and 24-hour recall data for Jamaican adults**.

Bland-Altman plots were used to assess the overall agreement between energy and macronutrient intakes measured by the FFQ and 24-hour recalls are presented in Figures 1, 2, 3 and 4. The plots suggest that the methods were comparable as no more than 4 of the observations were outside the limits of agreement for energy and the three macronutrients represented in the figures. Mean ratios of the FFQ to 24-hr recall values (95% CI) (antilog) were as follows: 1.16 (1.07-1.26), 0.97 (0.88-1.07), 1.40 (1.29 - 1.53), 1.05 (0.95 - 1.16), respectively for energy, protein, carbohydrate and fat. These data suggest that compared with estimates derived from the 24-hour recalls, on average the FFQ overestimated intakes of energy and carbohydrate but showed no real difference for intakes of protein and fat.

Cross-classification of the nutrients into quartiles showed that more than one-half of subjects were correctly classified into the same quartile of calcium, iron, folate, and alcohol intake; at least 40% were correctly classified for 10/18 nutrients investigated (Table 3). Gross misclassification into the opposite quartile was evident for protein, retinol and vitamin E. Weighted kappa values are also shown in Table 3 and ranged from 0.13 for retinol to 0.64 for alcohol. Moderate to good agreement (kappa above 0.4) was observed for monounsaturated fat, cholesterol, calcium, iron, folate, fiber and alcohol.

**Table 3:** Percentages of subjects classified into the same and opposite quartiles of nutrient intake (FFQ 1 and 24 hour recalls) and weighted Kappa (K _w_)

	Proportion (%) of subjects classified FFQ 1*		
	Agreement in same quartile	Misclassification in opposite quartile	Kappa (K _w_)
Total Energy (kcal)	48.5	10.0	0.40
Total Carbohydrate (g)	40.0	8.6	0.33
Protein (g)	32.8	24.3	0.17
Total Fat (g)	42.8	11.4	0.35
Total Saturated Fat	45.5	8.6	0.38
Total MUFA	41.4	7.1	0.42
Total PUFA	40.0	10.0	0.31
Linoleic	42.8	11.4	0.34
Linolenic	34.3	11.4	0.22
Cholesterol (mg)	47.1	7.1	0.43
Calcium (mg)	57.1	8.6	0.51
Iron (g)	51.4	10.0	0.48
Retinol (ug)	28.6	20.0	0.13
Beta-carotene (mg)	44.2	14.2	0.23
Vitamin E (mg)	32.8	23.8	0.15
Folate (g)	58.6	4.3	0.57
Total dietary fiber (g)	44.2	4.3	0.42
Alcohol	70.0	4.3	0.64

PUFA = polyunsaturated fatty acid. MUFA = monounsaturated fatty acid.

* Quartile by 24-hour recalls.

Table 4 shows the median intake of 9 food groups measured by the two FFQs and recalls. With the exception of intakes of cereals, poultry and fruits, the two FFQs gave similar median estimates of intakes of food groups Comparison of 24-hour recalls with the first and second administration of the FFQ showed similar intakes of food groups except for cereals, poultry, fruit and dark, green leafy and yellow vegetables. FFQ1 gave higher estimates of the consumption of cereals and fruits but lower intakes of poultry compared with recalls. FFQ2 also showed higher intakes of cereals when compared with recalls as well as higher intakes of poultry and green leafy and yellow vegetables. Intakes of fruits were lower when compared with recalls. Correlation coefficients between FFQ1 and 24-hour recalls were highest for eggs (0.56), dark, green leafy and yellow vegetables (0.53), red meat (0.55) and lowest for cereals (0.39), poultry (0.30) and fruits (0.27) (Table 4). In general, correlation coefficients between recalls and FFQ2 were higher than for FFQ1 ranging from 0.37 (poultry) to 0.61 (red meat).

**Table 4:** Daily median intakes of food groups (g day^-1^) estimated by the two food frequency questionnaires (FFQs) and twelve 24-hour recalls, and correlations between the two dietary methods ^a^

	Repeat 24- hour recalls Median	FFQ 1 Median	FFQ 2 Median	Spearman's *r *24-hr recalls *vs*. FFQ 1	Spearman's *r *24-hr recalls *vs*. FFQ 2
Cereals (g)	469.7	492.4	487.0 ^ab.^	0.39	0.42
Poultry (g)	48.1	36.6	55.1 ^ab^	0.30	0.37
Red meat (g)	16.9	14.1	15.2	0.55	0.61
Fish (g)	29.5	23.5	27.7	0.38	0.53
Eggs (g)	7.4	6.7	6.4	0.56	0.54
Fruits (g)	394.4	430.0	373.3 ^ab^	0.27	0.30
Dark green leafy and yellow vegetables (g)	85.8	89.0	97.9 ^b^	0.53	0.45
Other vegetables (g)	50.1	57.1	56.5	0.46	0.45
Peas and beans (g)	19.4	21.5	22.4	0.44	0.39

^a ^FFQ1 significantly different from FFQ 2.

^b ^FFQ intake significantly different from repeat recalls.

### Calibration - biomarkers

The validity of the FFQ to estimate intakes was also examined by dividing the sample into quartiles based on intakes from the FFQ and determining the mean level of biomarkers in each quartile (Table 5). Mean intakes of serum cholesterol and serum folate showed a significant trend of increasing mean level with increasing quartile; however a clear pattern was not observed for whole blood folate (Table 4). Partial correlation coefficients between the biomarker measurements and FFQ are also shown in Table 4. Serum cholesterol (unadjusted, *r *= 0.19; adjusted, *r *= 0.24) and serum folate (unadjusted, *r *= 0.22; adjusted, *r *= 0.25) were significantly correlated with dietary intakes. Without adjustment, whole blood folate was not significantly related to dietary intakes (*r *= 0.11) but with control for age, energy intake, BMI, current smoking and alcohol intake the partial correlation coefficient between dietary folate intake and blood concentrations was *r *= 0.33, p = 0.003.

**Table 5:** Comparison between biomarker levels and nutrient intakes estimated from the food frequency questionnaire according to quartiles and correlation coefficient between measures

	Levels in quartiles on the basis of FFQ				*p_trend_*	Correlation of biomarker with dietary intake^a^	
	Q1	Q2	Q3	Q4		unadjusted *r*	adjusted ^b ^*r*
Cholesterol							
Intake from FFQ (mg)	95.3 ± 38.9	179.4 ± 19.9	267.5 ± 30.9	468.9 ± 113.8	< 0.0001		
Serum cholesterol (*μ*mol/l)	4.46 ± 1.04	4.63 ± 0.96	4.69 ± 1.09	4.93 ± 1.07	0.020	0.19	0.24
Folate							
Intake from FFQ (*μ*g)	427 ± 102	643 ± 52	845 ± 63	1123 ± 138	< 0.0001		
Whole blood folate (ng/ml)	92.8 ± 132.1	108.0 ± 120.2	63.5 ± 33.4	94.9 ± 148.7	0.628	0.11 ^ns^	0.33
Serum folate (ng/ml)	9.55 ± 5.05	10.95 ± 3.95	11.15 ± 3.74	12.85 ± 5.84	0.009	0.22	0.25

^a ^With the exception of those indicated with ^ns ^not significant (p > 0.05), all correlations are significant at p < 0.01.

^b ^Adjusted for age, energy, BMI, current smoking,.

### Reproducibility

Reproducibility of the FFQ was determined by Intraclass correlations for unadjusted and energy-adjusted nutrient intakes comparing intakes obtained one year apart (Table 6). Unadjusted nutrient intake showed a high degree of reproducibility, ranging from *r *= 0.90 for iron to *r *= 0.71 for beta-carotene. With adjustment for energy intake, correlation coefficients ranged from 0.50 (saturated fat) to 0.84 (fiber) and tended to be lower than the unadjusted except for cholesterol, retinol, beta-carotene and vitamin E, vitamin K and fiber.

**Table 6:** Intraclass correlation coefficients for food frequency questionnaires completed at the beginning and end of 12 months^†^

	FFQ 1 vs. FFQ 2	
	Unadjusted *r *(CI)	Energy adjusted *r *(CI)
Total Energy (kcal)	0.87 (0.78, 0.92)	
Total Carbohydrate	0.85 (0.76, 0.91)	0.76 (0.61, 0.85)
Protein	0.83 (0.73, 0.90)	0.79 (0.66, 0.87)
Total Fat	0.85 (0.75, 0.90)	0.74 (0.57, 0.84)
Total Saturated Fat	0.78 (0.65, 0.87)	0.50 (0.18, 0.69)
Total MUFA	0.82 (0.71, 0.89)	0.79 (0.66, 0.87)
Total PUFA	0.75 (0.60, 0.80)	0.54 (0.24, 0.71)
Linoleic acid	0.75 (0.59, 0.85)	0.68 (0.48, 0.80)
Linolenic acid	0.73 (0.55, 0.83)	0.55 (0.26, 0.72)
Cholesterol	0.80 (0.67, 0.87)	0.80 (0.67, 0.87)
Calcium	0.85 (0.75, 0.91)	0.79 (0.66, 0.87)
Iron	0.90 (0.84, 0.94)	0.68 (0.47, 0.80)
Retinol	0.73 (0.56, 0.83)	0.74 (0.57, 0.84)
Beta-carotene (ug)	0.71 (0.53, 0.82)	0.75 (0.59, 0.85)
Vitamin E	0.77 (0.56, 0.82)	0.78 (0.58, 0.85)
Folate	0.88 (0.80, 0.92)	0.77 (0.62, 0.86)
Vitamin K	0.72 (0.54, 0.83)	0.72 (0.54, 0.83)
Zinc	0.79 (0.65, 0.87)	0.79 (0.66, 0.87)
Total dietary fiber	0.82 (0.70, 0.90)	0.84 (0.68, 0.90)
Alcohol	0.76 (0.45, 0.89)	0.68 (0.27, 0.86)

^a ^Energy-adjusted correlations between dietary methods as suggested by Willett (1)

^b ^All variables were log-transformed before analysis.

## Discussion

The paper describes the validity and reproducibility of a 120-item FFQ used to assess usual intake of nutrients consumed by Jamaican adults. Validity was assessed by comparing estimates obtained from the FFQ with those from multiple 24-h recalls in both genders in the general population and calibrated in men enrolled in a study of prostate cancer, using biological markers. The expanded FFQ performed consistently well in comparing energy and nutrient intakes (monounsaturated fat, cholesterol, calcium, iron, folate fiber and alcohol) estimated from 24-hour recalls with assessments by correlations, percentage correct classification and kappa statistics. Evaluation of intakes of food groups confirmed moderate relative validity. Calibration of the FFQ with biochemical markers of serum cholesterol and serum folate in cancer-free men showed modest correlations with coefficients similar to those obtained in other studies.

### Validity

#### Repeated recalls

Studies of the accuracy of the FFQ in measuring habitual intake are typically based on repeated 24-h recalls or daily weighed food intakes as reference measurements. In this study the relative validity of the instrument was evaluated against 24-hour recalls [2].

The expanded FFQ when compared to the earlier instrument generally showed stronger correlations with diet assessed by recalls. Energy-adjustment, a better measure of relative intake as adjustments may partially correct for measurement error [2], increased correlation coefficients for most macronutrients and micronutrients. However, adjustment for energy intake did not improve the coefficients for protein, linoleic acid, retinol, and vitamin E a phenomenon that occurs when variability is more related to systematic errors of under/overestimation than to energy intake [16,17]. Correlation coefficients between the second FFQ and recalls ranged from 0.31 to 0.86 (excluding retinol and vitamin E) and compared well with other studies [16-21]. Food intake estimated by the first FFQ showed moderate levels of relative validity compared with recalls. Correlation coefficients comparing 24-hour recalls and the first FFQ ranged from 0.27 - 0.56 and generally correlations were improved when compared with the second FFQ. Correlation coefficients were comparable to those observed in other validation studies [19,22-25]. Cereals and dark green leafy and yellow vegetables were most likely to be over-reported.

Weighted kappa statistics are recommended to evaluate the likely impact of measurement error and ĸ values above 0.4 are valid for conclusions [26]. The FFQ performed reasonably well (ĸ values, 0.41 - 0.64) for energy, monounsaturated fat, cholesterol, calcium, iron, folate, fiber, alcohol and was fair for other nutrients. Similar to correlational analyses and the percentages classified / misclassified, the weighted kappa results indicate that the FFQ did not adequately classify subjects with respect to protein, retinol and vitamin E intake levels suggesting that intakes of these nutrients were not well assessed by the FFQ.

The Bland-Altman method assesses agreement between two measurement methods [27] and was used determine the bias and limits of agreement (LOA) for estimates of energy and macronutrient intakes. Distribution of points within the limits of agreement suggested that the FFQ and 24-hour recall methods were comparable although the mean and interval estimates for bias indicated that the FFQ over-estimated energy and carbohydrate intakes. Comparison of results for energy and macronutrients (log-transformed) with other studies showed that the LOAs were narrower than those reported in the Shanghai Men's Health Study (China) [25] and the Blue Mountains Eye Study in Australia [28].

Bland-Altman plots showed that the estimates of energy and macronutrient intakes (excluding carbohydrate) obtained by the FFQ were comparable to those from repeated dietary recalls and unlikely to cause systematic biases. While the kappa statistic for protein suggested poor agreement between FFQ and 24-hr recall for protein intake, the Bland-Altman plots suggested that the two tools were comparable for this nutrient. This implies that, while the FFQ will give similar results to the 24-hour recall for continuous measures of protein intake, both tools may not lead to agreement on classification by quartile. For the other macronutrients, both methods of assessing agreement do concur. Correlation analyses suggest that for all nutrients excepting vitamin E, retinol and protein and total PUFA, there is a relatively high positive linear correlation between energy adjusted nutrients intake by FFQ and that obtained from 24-hr recall. Thus, high values from FFQ are associated with high values from the 24-hr recall.

#### Biomarkers

Sources of errors for biomarkers were uncorrelated with those for the FFQ or other dietary methods. Biomarkers are assumed to be responsive to dietary intakes in a dose-dependent manner, nonetheless, they may not necessarily reflect long-term dietary intake and are influenced by genetic and environmental factors; correlation coefficients tend to be low [2]. In this study whole blood / serum folate and serum cholesterol were used as a second measure of the validity of the FFQ in view of their hypothesized relationship with cancer [29,30]; other nutrients were not investigated due to resource constraints.

A dose-response relationship between dietary intake and serum cholesterol was observed and correlation coefficients were modest (unadjusted, *r *= 0.19; adjusted, *r *= 0.24). These results are better than those of Katsouyanni et al's study of Greek schoolteachers which observed negative correlations between first and second administration of the FFQ and recalls and plasma concentrations of cholesterol (*r *= -0.07, *r *= -0.09, and *r *= -0.00, respectively) [31]. Although there are methodologic differences in the measurement of dietary intakes, Newby et al reported a significant and inverse correlation between the revised Diet Quality Index (DQI) and plasma concentrations of total cholesterol (*r *= -0.22; *p *< 0.05) which suggested that low cholesterol levels were associated with a good diet [32].

Serum folate but not whole blood folate increased with increasing levels of dietary intakes. Whereas serum folate was positively related to dietary intakes, unadjusted correlation between folate intakes estimated with the FFQ and whole blood folate was weak and non significant. These differences in agreement may be related to the sensitivity of serum and whole blood folate as biomarkers of diet. Serum folate is considered to reflect recent folate intake whereas blood folate is suggestive of long-term intake [33]. Correlation coefficients of serum folate and dietary folate intakes in this study were higher than van de Rest et al's study that measured folate intake over the previous 3 months in elderly Dutch people (*r *= 0.14) [34] but lower than others [35-37]. Weinstein et al reported that Healthy Eating Index scores were positively correlated with serum folate (r = 0.25) [38]. With control for potential confounders, dietary folate intake was significantly correlated with whole blood folate in the present study (*r *= 0.33) and was similar to that reported by McDonald et al's study of post-menopausal women (*r *= 0.30) [39]. Some studies comparing dietary intake with erythrocyte folate have reported coefficients that were higher (*r *= 0.55 - 0.67) [21,40,41] while in others correlations were weak or failed to show an association [34,37,42-44]. Thus our data suggest that the FFQ provides a valid measure of folate and cholesterol intake in diet and disease associations.

### Reproducibility

The reproducibility of the FFQ administered one year apart was good with reasonably high correlation coefficients (0.50 to 0.88) that were similar to [16,17,20,21] or higher than reported by others [25]. In the previous FFQ, correlation coefficients were generally lower (0.42 to 0.71) than observed in the expanded instrument although both were re-administered after one year [2]. It is possible that the expanded FFQ produces a better estimate of usual intake.

### Strengths / limitations

The validity of the expanded FFQ was evaluated by repeat 24-hour recalls and biomarkers and several different statistical tests were used to evaluate associations between the methods. Similar to other epidemiologic studies, a single blood collection was used for comparison with habitual intake estimated by the FFQ. The use of additional biomarkers would be ideal for the calibration study, however, resource constraints did not allow for further investigations. A single interviewer conducted the dietary recalls which should have minimized variation in administration.

Absolute intake estimates of FFQ-derived nutrients were higher for most nutrients when compared to 24-hour recalls, the reference method. Some food items on the FFQ may not have been consumed during any of the dietary recalls and this may have contributed to the difference. For example, we included an extensive listing of beans and legumes to address a subsidiary objective of the study. These foods were rarely consumed and may have attenuated validation coefficients. Notwithstanding, FFQs have been reported to yield higher estimates for most nutrients than reference methods [7,8,17,20,31,45].

## Conclusions

Our results indicate that the relative validity (compared with recalls) of the expanded FFQ was good in estimating food groups and nutrients (with the exception of retinol and vitamin E) and acceptable for the investigation of associations of diet with risk of disease. Serum levels of cholesterol and folate correlated with dietary intakes and illustrate the feasibility of using these biomarkers for exposure assessment in studies of diet and disease. We conclude that the FFQ is a useful dietary assessment tool in the Jamaican population for food groups and most nutrients of interest in assessment of dietary associations with cardiovascular diseases and cancer. However, for a few nutrients this methodology is unlikely to provide an adequate measure of habitual intake.

## Competing interests

The authors declare that they have no competing interests.

## Authors' contributions

All authors contributed to the design and the execution of the study. MJ conducted the analysis and drafted the manuscript. NY contributed to statistical analyses and interpretation of analytic results. All authors critically reviewed the manuscript.

## Appendix 1

### List of food and drink items included in the expanded Jamaican food frequency questionnaire

Rice and peas, brown rice, white rice, whole wheat bread, white bread, hard-dough bread, boiled dumpling, fried dumplings, cornmeal porridge, oatmeal porridge, other porridges, ready-to-eat cereals, biscuits/crackers, macaroni/ spaghetti, yam, green banana, fried plantain, sweet potato, Irish potato, breadfruit, egg, fried chicken, roasted / baked chicken, mutton, pork, beef, fresh fish, shell fish, chicken back, corned beef, ham / bacon, sausage, liver, canned fish, tuna, pickled mackerel, salted fish, pig's tail, red peas, gungo peas, chick peas, baked beans, broad bean, lentils, soy beans, black beans, black-eye peas, split peas, peanuts, peanut butter, other nuts, orange, papaya, mango, ripe banana, pineapple, grape, American apple, melon, Otaheti apple, orange juice, other fruit juices, osther vegetable juice, callalo, ochro, carrot juice, carrot, peas and carrot, pumpkin, pakchoi, string bean, cabbage, tomato, sweet pepper, vegetable salad, avocado, ackee, beet, cucumber, corn, broccoli, cauliflower, sweetened condensed milk, evaporated milk, soy milk, whole milk powder, skimmed milk power, liquid whole milk, liquid skimmed milk, flavoured milk, Supligen, cheddar cheese, processed cheese, ice-cream, cake / cookies, bun/ bulla, chips, butter, margarine, vegetable patty, meat patty, hamburger, hotdog, pizza, ketchup, jam / jelly, sugar, hard candies, chocolate, coffee, tea, cocoa, malta, beer /stout, rum, red wine, white wine, soda, syrup drink, herbal tea.
